# Consolidation Differentially Modulates Schema Effects on Memory for Items and Associations

**DOI:** 10.1371/journal.pone.0056155

**Published:** 2013-02-07

**Authors:** Marlieke T. R. van Kesteren, Mark Rijpkema, Dirk J. Ruiter, Guillén Fernández

**Affiliations:** 1 Donders Institute for Brain, Cognition and Behaviour, Radboud University Nijmegen, Nijmegen, The Netherlands; 2 Department of Anatomy, Radboud University Nijmegen Medical Centre, Nijmegen, The Netherlands; 3 Department of Cognitive Neuroscience, Radboud University Nijmegen Medical Centre, Nijmegen, The Netherlands; CSIC-Univ Miguel Hernandez, Spain

## Abstract

Newly learned information that is congruent with a preexisting schema is often better remembered than information that is incongruent. This schema effect on memory has previously been associated to more efficient encoding and consolidation mechanisms. However, this effect is not always consistently supported in the literature, with differential schema effects reported for different types of memory, different retrieval cues, and the possibility of time-dependent effects related to consolidation processes. To examine these effects more directly, we tested participants on two different types of memory (item recognition and associative memory) for newly encoded visuo-tactile associations at different study-test intervals, thus probing memory retrieval accuracy for schema-congruent and schema-incongruent items and associations at different time points (t = 0, t = 20, and t = 48 hours) after encoding. Results show that the schema effect on visual item recognition only arises after consolidation, while the schema effect on associative memory is already apparent immediately after encoding, persisting, but getting smaller over time. These findings give further insight into different factors influencing the schema effect on memory, and can inform future schema experiments by illustrating the value of considering effects of memory type and consolidation on schema-modulated retrieval.

## Introduction

Information that is congruent with prior knowledge (or a *schema)* is often found to be better remembered than incongruent information [1], [2]. This *congruency effect or schema effect* on memory is suggested to be dependent on mnemonic mechanisms [3], [4], such as differentially efficient encoding [5], [6] and consolidation processes [7]–[9]. However, the relative contribution of these processes to schema-dependent memory enhancement is largely unknown. Moreover, reports of enhancing schema effects on memory are not always consistent in the literature, as not all types of memory appear to be enhanced by a pre-existing schema [10], [11], suggesting that the way a memory is cued can influence the schema effect as well. Thus, the relative contribution of encoding and consolidation processes on the schema effect and their enhancing effects on different memory measures still remains to be established.

The schema effect on memory has been a fairly consistent finding for decades, showing that information that fits with a pre-existing schema is better remembered [2], [12], and more efficiently processed [5], [6]. However, opposing observations where schema-inconsistent memories are shown to be enhanced are also occasionally reported [10]. These paradoxical effects are generally related to detailed recognition [13], interference effects [14], false memories and confidence [15], [16], and category learning [11], [13], and are largely consistent with the *novelty encoding* principle stating that information that is novel is preferentially encoded [17]. Partly as a result of these seemingly contradictory results, the schema theory was rendered more labile over the past decades [10]. As learning of congruent information does not always consistently lead to better memory performance than incongruently learned information, it was suggested that the schema effect might be dependent on various additional factors, such as how a memory is cued and after which delay it is measured [4], [11]. These additional factors might account for the paradoxical effects of a schema on memory performance that are mentioned above.

During memory encoding, a new memory trace is processed such that it can be most efficiently stored [18]. Encoding is suggested to be dependent on many factors, such as depth of processing [19] and semantic elaboration [20], processes that are found to be enhanced when an encoded stimulus is congruent with prior knowledge [21]. However, also novelty is suggested to drive (presumably different [4]) encoding processes [17], leading stimuli incongruent with prior knowledge to be better encoded as well. After encoding, a memory is thought to be integrated into existing knowledge structures through consolidation mechanisms [9], [22], [23], which are proposed to process memory traces off-line in order to most efficiently assimilate them into preexisting schemas [24], [25]. This consolidation process is found to be facilitated specifically for information that was related to a pre-existing schema [8], [12], and might additionally be related to tagging of a schema-related memory during and right after encoding [26]. The ease and nature of retrieval of a certain memory is thus suggested to depend not only on how a memory trace is encoded but also on how it is consolidated and integrated into the preexisting schema [3], [27]. Consolidation of a memory after encoding is moreover found to favor strengthening of salient and important memories, such as memories that are emotional [28], rewarding [29], or semantically related [30], thus suggesting that consolidation, next to encoding, can have profound effects on long-term storage of a memory trace [24].

**Figure 1:**
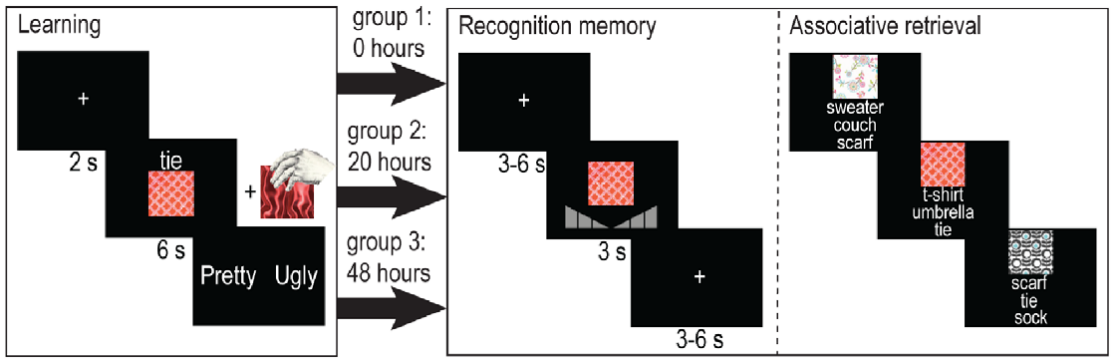
Experimental design. On day 1, participants learned associations of visual motifs and congruent or incongruent object-fabric combinations, where the object was presented together with the motif as a written word on the computer screen, and the fabric simultaneously as a tactile stimulus underneath the computer screen. Participants were tested after different time intervals (group 1: t = 0 hours, group 2: t = 20 hours, group 3: t = 48 hours) by means of a visual item recognition test (motifs) and an associative memory test in which the motifs served as cues and the associated word was asked for in a three-choice test.

In this experiment, we therefore examined how the congruency effect on memory progresses over time by examining memory performance before or after consolidation on retrieval of congruent versus incongruent item and associative memories. Participants were randomly divided in three different groups (delay t = 0 hours, t = 20 hours (as described in [12]), or t = 48 hours after encoding), and were tested using a between-subjects 3×2 factorial design with study-test interval (delay) and congruency as factors. They completed a paradigm in which they learned visuo-tactile associations that were either congruent or incongruent with prior knowledge (see [12] and figure 1), and performed memory tests either after 0 hours (group 1), after 20 hours (group 2) or after 48 hours (group 3). They were first tested on item recognition and subsequently on associative memory. Analyses were conducted on both these memory measures and compared for all three groups. We expected the schema effect to be apparent for both item recognition and associative memory scores, but hypothesized that differences could arise over time, through consolidation.

## Results

**Figure 2:**
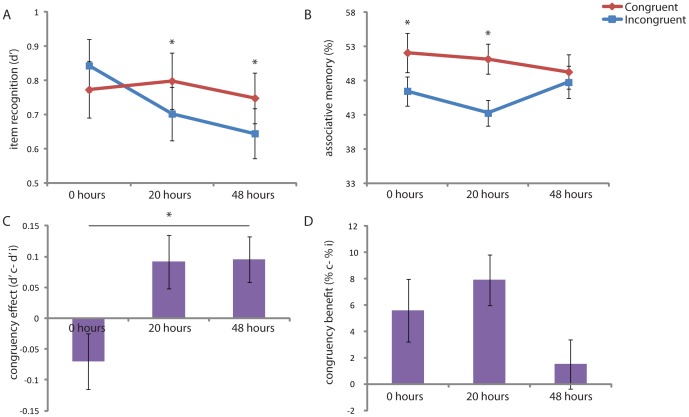
Behavioral results. Item recognition scores (d’) for schema-congruent memories were enhanced only after consolidation (A), while schema-congruent associative memory scores (% correct) were enhanced already immediately after encoding and this effect persisted during time (B). Panels C and D show the congruency effect for both these memory measures over time, where the congruency effect on memory is found to increase for item recognition (C), but not for associative memory (D).

Item recognition memory scores (d-prime, figure 2A) showed a delay x congruency interaction (F(2,66) = 5.04, p<.01), with item memory significantly better for congruent items in group 2 (t(22) = 2.12, p<.05), and group 3 (t(22) = 2.55, p<.05), but not in group 1 (t(22) = 1.55, p = n.s.). No main effect of congruency was found (F(2,66) = 2.53, p = n.s.). All measures were significantly different from chance (group 1 congruent: t(22) = 9.42, p<.001, group 1 incongruent: t(22) = 10.92, p<.001, group 2 congruent: t(22) = 9.97, p<.001, group 2 incongruent: t(22) = 9.32, p<.001, and group 3 congruent: t(22) = 10.32, p<.001, group 3 incongruent: t(22) = 8.85, p<.001). Reaction times did not show any differences for either group (group 1: t(22) = .44, p = n.s., group 2: t(22) = .52, p = n.s., group 3: t(22) = .12, p = n.s.), or between-groups (congruent: F(1,66) = .31, p = n.s., incongruent: F(1,66) = .36, p = n.s., also not in any post-hoc analyses). These results show a delay x congruency interaction for item recognition memory scores based on a schema effect that arises only after a delay that allows consolidation processes to take place (figure 2A).

Associative memory scores (figure 2B) showed a main effect of congruency (F(1,66) = 17.59, p<.001) without a delay x congruency interaction (F(2,66) = 2.44, p = n.s.). Also all these measures were significantly different from chance (group 1 congruent: t(22) = 6.58, p<.001, group 1 incongruent: t(22) = 6.18, p<.001, group 2 congruent: t(22) = 8.16, p<.001, group 2 incongruent: t(22) = 5.30, p<.001, and group 3 congruent: t(22) = 6.39, p<.001, group 3 incongruent: t(22) = 6.16, p<.001). Associative memory scores thus show a main effect of congruency and no interaction with delay (figure 2B).

Assessing the strength of the congruency effect (congruent – incongruent) over time (delay) (figure 2C and 2D) showed a significant positive increasing relationship for item recognition (t(2) = 2.75, p<.01, congruent>incongruent, figure 2C), which was significant for group 2> group 1 (t(44) = 2.57, p<.05) and group 3> group 1 (t(44) = 2.82, p<.01), but not for group 3> group 2 (t(44) = −.07, p = n.s.). For associative memory (figure 2D), this analysis did not reveal a significant delay × congruency effect interaction (t(2) = −1.38, p = n.s.). Thus, a delay × congruency effect interaction was only found for item recognition, where the congruency effect was found to become larger over time.

## Discussion

The results reported here show that encoding and consolidation differentially affect the schema effect on memory for different memory types. By testing the schema effect for both item recognition and associative memory at different delays after learning, we show that the schema effect for item recognition increases with consolidation, while not yet being apparent immediately after encoding. On the other hand, for associative memory the schema effect is found to be present already after encoding, and, although the difference grows smaller, shows persistence over consolidation. These results show that the schema effect on memory depends on delay and type of memory test.

These results are generally consistent with the schema theory [1], while the finding that the schema effect only arises after consolidation for item recognition additionally partly accounts for findings that are inconsistent with it [11]. Schema theory has gained a challenging character over the past decades [10] because of paradoxical findings that showed enhanced memory for either information congruent or incongruent with a pre-existing schema. Schema effects were therefore suggested to be dependent on several factors [4], [11], of which two were specifically tested here. We believe our findings along with previous inconsistencies in the literature can partly be explained by (schema) consolidation theories [3], [4], [31], stating that schema-congruent memories are preferentially consolidated in an accelerated manner, and its effects on memory performance over time for both item recognition and associative memory measures. Therefore, we propose that future research on schema-dependent memory should take these modulatory factors into account.

Schema effects have thus been suggested to be highly dependent on the specific task at hand. For example, while we report schema effects on item recognition, this enhancing effect is not always consistently found. When item recognition is tested in a two-alternative-forced-choice (2AFC) task where participants are instructed to choose between the target and a highly similar lure items that are incongruent with a pre-existing schema are found to be better remembered [11], [13]. Moreover, these results are generally reported when tested immediately after learning without consolidation. While our results show no significant effect of schema on item recognition immediately after learning (figure 2), they do show an interaction with performance over a delay, when allowing consolidation processes to take place. This suggests that enhancing effects of incongruent memories right after encoding could inverse after consolidation has taken place, favoring schema-consistent memories in the long run only [24], [30]. Additionally, the incongruency of a memory trace might lead to novelty and saliency processes that possibly preferentially enhance short-term storage of the memory [4], [17]. Therefore, schema-inconsistent memory enhancements e.g. in the 2AFC task would benefit from future research where retrieval tests are performed both before and after consolidation, to more specifically determine whether this effect is specifically related to encoding mechanisms and to better understand its relation to consolidation mechanisms. Other factors determining schema-congruent and schema-incongruent memory enhancements, such as the type of task, type of cue, and confidence could profit in the same way when future research will more clearly distinguishes between encoding and consolidation effects.

In sum, these findings give more insight into two different factors that modulate schema effects on memory: memory type and consolidation. Results show that the schema effect on item recognition performance is mostly influenced by consolidation processes occurring after learning, while the schema effect on associative memory is already present immediately after encoding and persists after consolidation. These results thus demonstrate that schema effects on memory performance can be more complex than previously thought since they are affected both by the type of cue during retrieval and the degree of consolidation that passed before retrieval. Further research will need to examine the specifics of this phenomenon, both behaviorally and neurally.

## Materials and Methods

### Participants

This experiment is an extension of a previously published experiment [12], which is taken along in the analysis reported here (group 2). Stimuli, design, and procedures are exactly the same as reported in this previous study. Seventy-six native Dutch female right-handed students participated in this study. All were healthy and had normal or corrected-to-normal vision. They were paid to participate and were told that they could earn extra money for better performance. Participants were randomly assigned to either group (with delay t = 0 hours (group 1), delay t = 20 hours (group 2) or t = 48 hours (group 3) between encoding and retrieval), with 26 participants in group 1 and 25 participants in group 2 and 3. Seven participants (3 in group 1 and 2 in group 2 and 3) were excluded after data acquisition, because of poor item memory performance (total item recognition hits <30), which left 69 (23 per group) participants for analyses. This sample covered an age range of 18–33 years, with a mean age of 22.14 years. There were no age differences between the different groups (group 1∶22.48, group 2∶22.65, group 3∶21.30, F = 1.106, p = n.s.). Participants in group 2 self-reported to have slept on average 7.67 (range 6–9) hours in the night after learning and participants in group 3 self-reported to have slept on average 7.22 hours (range 2.5–10) in the night after learning and on average 7.46 hours (range 5.5–10) in the night before testing. Hours of sleep was not significantly different between these groups for the first night (t = 1.26, p = n.s.). We decided to recruit women only, because they generally have more interest in and knowledge about fashion-like stimuli, and they are shown to have more tactile spatial acuity in their fingertips than men [32]. Ethical approval was obtained from the institutional review board (CMO Region Arnhem-Nijmegen, The Netherlands), and all participants gave written informed consent.

### Stimuli

Participants learned a series of triplets of simultaneously presented stimuli that, when associated with each other, formed an object likely to be present in real life [12]. These associations consisted of 1) *motifs* (200), visually presented as a 2-dimensional, pictorial square without tactile information; 2) visually presented *object words* (20) describing objects primarily composed of fabrics; and 3) *fabric samples* (20) that could be linked to the object words. Motifs (400 in total, including lures) were obtained from the internet, and were equalized in size (256×256 pixels, 28.35 pixels/cm, indexed color mode) and auto contrasted using Adobe Photoshop CS3, version 10.0.1 (Adobe, San Jose, CA, USA). Fabric samples were cut into squares of five by five cm, and object-fabric combinations were categorized as being either semantically congruent (for example a leather jacket) or semantically incongruent (for example a lace umbrella). The (in) congruency of these combinations was verified in an independent behavioral pilot, where participants (n = 12) were asked to rate the congruency of word-fabric combinations from 1–6. Combinations rated on average 2.5 or lower were considered incongruent, and combinations rated on average 3.5 or higher were considered congruent. Combinations in between these ratings were altered to either fit a congruent or incongruent representation.

### Design and General Procedure

Participants were all tested using the same procedure, with the only dependent variable the delay between encoding and retrieval (0 hours for group 1, 20 hours for group 2 and 48 hours for group 3). They were tested using two (one for item recognition and one for associative memory) within-subjects 2×2 factorial designs with congruency (congruent items versus incongruent items) and memory (associatively remembered items versus associatively forgotten but item remembered items and item remembered versus completely forgotten items) as within-subject factors [see figure 1 and 12], and were subsequently tested in a between-subjects design with different study-test delay (group 1 versus group 2 versus group 3). They were invited to come to the center on one (group 1) or two days (group 2 and 3) with 48 hours between the two visits. On day one, participants were instructed to memorize simultaneously presented triplets of visual motifs, visual object words, and tactile fabric samples by imagining how the combination of these features would look like. They were told that their memory would be tested either directly after (group 1) on the next day (group 2 and 3), but they received no information about the specifics of this memory test. Using Presentation 10.2 (Neurobehavioral systems, Albany, CA, USA), the motif and the word were visually presented on a computer screen for six seconds, the word situated above the motif. Concurrently, participants were instructed in a practice session to tactilely explore a fabric for the complete 6 seconds, and imagine how the combination of motif, word, and fabric would look. The fabric was presented by the experimenter underneath a heightened plateau on which the computer screen was placed, and was not visible to the participant. After presentation of each stimulus combination, participants were asked to indicate whether they thought the triplet characterizing the imagined object was either pretty or ugly (see figure 1). After encoding, participants in group 1 was tested while participants in group 2 and 3 went home and returned to the center respectively 20 or 48 hours later.

In total, participants memorized 200 sequentially presented combinations, 100 congruent and 100 incongruent, divided into three sessions of consecutively 80, 80, and 40 trials. Because the object words and fabric samples had to be divided equally for each session and each condition, the 20 object words and 20 fabric samples were combined into 80 possible combinations (40 congruent and 40 incongruent), so each object word and each fabric sample was linked to two congruent and two incongruent fabrics. Within each session, these 80 object-fabric associations were randomly divided, but equal for each participant, whilst motifs were randomly shuffled for each participant and thus unique for each combination. For the last session of 40 presentations only one congruent and one incongruent object-fabric combination was used instead of two. Thus, every participant learned the same object-fabric combinations, but for each participant these were differently associated with the motifs.

During retrieval, participants performed an item recognition memory test (with confidence rating) for the motifs presented the day before. They were instructed to respond within the three seconds presentation time. Participants received a practice session before starting the experiment. Stimuli were presented in the center of the screen for three seconds, and were followed by a fixation cross, presented for three to six seconds. Furthermore, 10 fixation cross baseline trials of 10 seconds duration were included. These baseline trials were distributed so that within every 40 trials, a baseline trial was presented. The item recognition memory test lasted in total 51 minutes and 20 seconds. After, participants performed an associative retrieval task additionally.

### Memory Tests and Analyses

Item recognition memory was tested using a confidence level approach (6 levels) in which participants were instructed to indicate whether a perceived stimulus (200 old and 200 new) was old or new. Six answer options were provided: sure old, nearly sure old, not sure old, not sure new, nearly sure new and sure new. The order of the motifs was pseudorandom; no more than four consecutive old or new stimuli were presented. Participants could only answer once and were given feedback on which button they pressed. Answers that were given too late (i.e. after the three seconds presentation time), or were indicated as not sure, were not included in the analyses.

Subsequent to the item recognition memory test, participants performed a self-paced, three-alternative forced-choice associative memory task, in which they were instructed to indicate which object word was associated with a certain motif on the previous day. All 200 memorized motifs were randomly and sequentially presented on a computer screen as cues, together with three words of which one word was the correct answer, and the two other words were randomly sampled from the other 19 words. After participants finished this test, they filled out a study-specific questionnaire.

Behavioral measures of item recognition scores were analyzed using SPSS 15.0 (SPSS Inc., Chicago, IL, USA) by calculating the percentage of hits and false alarms (both sure old and nearly sure old confidence levels) for both conditions (congruent and incongruent). Next, these values were z-transformed and subtracted from each other to calculate d-prime for both conditions. Subsequently, a repeated measures ANOVA with the factors time (group 1, group 2, and group 3) and congruency (congruent versus incongruent) were performed to test interactions and main effects between these factors. Group effects on single measures were conducted using a one-way ANOVA. For post-hoc analyses, Student t-tests were performed to determine differences from chance level (0; one-sample t-test) and differences between the congruent and incongruent conditions within both groups (paired-samples t-test), and differences between groups (independent-samples t-test). Associative memory was analyzed using only the items that were correctly recognized during item recognition. Of these items, percentage correct was calculated for both conditions, and again tested using a one-sample (with chance level 1/3) and again tested using a repeated measures ANOVA and subsequent paired samples and independent samples Student t-tests, as described above. Congruency effects were calculated per group and per memory type by subtracting individual incongruent memory scores from congruent memory scores (so congruent – incongruent) and were subsequently tested using a linear regression analysis. Also reaction time differences between both congruency conditions were assessed using the same statistical tests. Alpha was set at.05 throughout.

## References

[1] Bartlett FC (1932) Remembering: a study in experimental and social psychology. Cambridge, [England]: University Press. 317p.

[2] Bransford JD, Johnson MK (1972) Contextual prerequisites for understanding - some investigations of comprehension and recall. Journal of Verbal Learning and Verbal Behavior 11: 717–726.

[3] Wang SH, Morris RG (2010) Hippocampal-neocortical interactions in memory formation, consolidation, and reconsolidation. Annual Review of Psychology 61: 49–79, C41–44. 10.1146/annurev.psych.093008.100523 19575620

[4] van Kesteren MT, Ruiter DJ, Fernandez G, Henson RN (2012) How schema and novelty augment memory formation. Trends in Neurosciences 35: 211–219. 22398180 10.1016/j.tins.2012.02.001

[5] Tse D, Takeuchi T, Kakeyama M, Kajii Y, Okuno H, et al (2011) Schema-dependent gene activation and memory encoding in neocortex. Science 333: 891–895. 21737703 10.1126/science.1205274

[6] van Kesteren MT, Fernandez G, Norris DG, Hermans EJ (2010) Persistent schema-dependent hippocampal-neocortical connectivity during memory encoding and postencoding rest in humans. Proceedings of the National Academy of Sciences of the United States of America 107: 7550–7555. 20363957 10.1073/pnas.0914892107 PMC2867741

[7] Squire LR (1992) Memory and the hippocampus: a synthesis from findings with rats, monkeys, and humans. Psychological Review 99: 195–231. 1594723 10.1037/0033-295x.99.2.195

[8] Tse D, Langston RF, Kakeyama M, Bethus I, Spooner PA, et al (2007) Schemas and memory consolidation. Science 316: 76–82. 17412951 10.1126/science.1135935

[9] Frankland PW, Bontempi B (2005) The organization of recent and remote memories. Nature Reviews Neuroscience 6: 119–130. 15685217 10.1038/nrn1607

[10] Alba JW, Hasher L (1983) Is Memory Schematic. Psychological Bulletin 93: 203–231.

[11] Sakamoto Y, Love BC (2004) Schematic influences on category learning and recognition memory. J Exp Psychol Gen 133: 534–553. 15584805 10.1037/0096-3445.133.4.534

[12] van Kesteren MT, Rijpkema M, Ruiter DJ, Fernandez G (2010) Retrieval of associative information congruent with prior knowledge is related to increased medial prefrontal activity and connectivity. Journal of Neuroscience 30: 15888–15894. 21106827 10.1523/JNEUROSCI.2674-10.2010 PMC6633736

[13] Davis T, Love BC, Preston AR (2012) Learning the exception to the rule: model-based FMRI reveals specialized representations for surprising category members. Cerebral Cortex 22: 260–273. 21666132 10.1093/cercor/bhr036

[14] Anderson JR (1981) Effects of Prior Knowledge on Memory for New Information. Memory & Cognition 9: 237–246.

[15] Rojahn K, Pettigrew TF (1992) Memory for schema-relevant information: a meta-analytic resolution. Br J Soc Psychol 31 (Pt 2): 81–109. 10.1111/j.2044-8309.1992.tb00958.x 1535823

[16] Neuschatz JS, Lampinen JM, Preston EL, Hawkins ER, Toglia MP (2002) The Effect of Memory Schemata on Memory and the Phenomenological Experience of Naturalistic Situations. Applied Cognitive Psychology 16: 687–708.

[17] Tulving E, Kroll N (1995) Novelty Assessment in the Brain and Long-Term-Memory Encoding. Psychonomic Bulletin & Review 2: 387–390. 24203720 10.3758/BF03210977

[18] Paller KA, Wagner AD (2002) Observing the transformation of experience into memory. Trends Cogn Sci 6: 93–102. 15866193 10.1016/s1364-6613(00)01845-3

[19] Craik FIM, Lockhart RS (1972) Levels of Processing - Framework for Memory Research. Journal of Verbal Learning and Verbal Behavior 11: 671–684.

[20] Craik FIM, Tulving E (1975) Depth of Processing and Retention of Words in Episodic Memory. Journal of Experimental Psychology-General 104: 268–294.

[21] Staresina BP, Gray JC, Davachi L (2009) Event congruency enhances episodic memory encoding through semantic elaboration and relational binding. Cerebral Cortex 19: 1198–1207. 18820289 10.1093/cercor/bhn165 PMC2665161

[22] Squire LR, Alvarez P (1995) Retrograde amnesia and memory consolidation: a neurobiological perspective. Current Opinion in Neurobiology 5: 169–177. 7620304 10.1016/0959-4388(95)80023-9

[23] Walker MP, Stickgold R (2004) Sleep-dependent learning and memory consolidation. Neuron 44: 121–133. 15450165 10.1016/j.neuron.2004.08.031

[24] Lewis PA, Durrant SJ (2011) Overlapping memory replay during sleep builds cognitive schemata. Trends in Cognitive Sciences 15: 343–351. 21764357 10.1016/j.tics.2011.06.004

[25] Diekelmann S, Born J (2010) The memory function of sleep. Nat Rev Neurosci 11: 114–126. 20046194 10.1038/nrn2762

[26] Redondo RL, Morris RG (2011) Making memories last: the synaptic tagging and capture hypothesis. Nature Reviews Neuroscience 12: 17–30. 21170072 10.1038/nrn2963

[27] Dudai Y (2012) The restless engram: consolidations never end. Annual Review of Neuroscience 35: 227–247. 10.1146/annurev-neuro-062111-150500 22443508

[28] Payne JD, Kensinger EA (2011) Sleep leads to changes in the emotional memory trace: evidence from FMRI. Journal of Cognitive Neuroscience 23: 1285–1297. 20521852 10.1162/jocn.2010.21526

[29] Fischer S, Born J (2009) Anticipated reward enhances offline learning during sleep. Journal of Experimental Psychology Learning, Memory, and Cognition 35: 1586–1593. 10.1037/a0017256 19857029

[30] Payne JD, Tucker MA, Ellenbogen JM, Wamsley EJ, Walker MP, et al (2012) Memory for semantically related and unrelated declarative information: the benefit of sleep, the cost of wake. PLoS One 7: e33079. 22457736 10.1371/journal.pone.0033079 PMC3310860

[31] McKenzie S, Eichenbaum H (2011) Consolidation and reconsolidation: two lives of memories? Neuron 71: 224–233. 21791282 10.1016/j.neuron.2011.06.037 PMC3145971

[32] Peters RM, Hackeman E, Goldreich D (2009) Diminutive digits discern delicate details: fingertip size and the sex difference in tactile spatial acuity. J Neurosci 29: 15756–15761. 20016091 10.1523/JNEUROSCI.3684-09.2009 PMC3849661

